# Wire Injury-Induced Moderate Aortic Valve Stenosis in Mice Is Accompanied by a Chronic Systemic Inflammatory Reaction

**DOI:** 10.3390/cells14120883

**Published:** 2025-06-11

**Authors:** Katrin Becker

**Affiliations:** 1 Cardiovascular Research Laboratory, Department of Cardiology, Pulmonary Diseases and Vascular Medicine, Medical Faculty, University Hospital Düsseldorf, Heinrich-Heine University, 40225 Düsseldorf, Germany; katrin.becker@uni-duesseldorf.de; 2 Institute for Molecular Cardiology, Medical Faculty, University Hospital Düsseldorf, Heinrich-Heine University, 40225 Düsseldorf, Germany; 3 Faculty of Medicine, University Hospital Cologne, University of Cologne, 50937 Cologne, Germany

**Keywords:** cardiac remodelling, costimulatory molecules, heart function, lymphocyte activation, T cells

## Abstract

Background/Objectives: While the presence of inflammatory processes in stenotic aortic valves is acknowledged, no systematic characterization of the systemic immune reaction upon aortic valve stenosis (AS) has been performed yet. The hypothesis of this study was that AS induces a systemic inflammatory reaction linked with local processes in the heart. Methods: Murine wire injury (WI) to induce AS, or sham surgery, were performed prior to the 4-week assessment of AS severity, left ventricular (LV) function and hypertrophy with echocardiography (echo). Organ weights, levels of leukocytes, cytokines and costimulatory molecules in blood, heart, and peripheral immune organs (spleen, liver, lymph nodes), and immune cell uptake of Cy5-labelled perfluorocarbon nanoemulsions were measured. Results: Trends towards correlation were found between organ weights, myocardial immune cells and echo. Cytokine mRNA levels trended mainly towards an increase in heart and regional lymph nodes and a reduction in spleen and liver, and correlation with echo was more homogeneous after WI. Unchanged cytokine protein levels in myocardium and plasma trended to correlate with echo. A homogeneous pattern was found for echo and costimulatory molecule correlation, while PFC uptake by lymphatic cells was reduced upon AS. Conclusions: The results suggest a link between number and activation state of leukocytes in peripheral organs and cardiac processes in AS. Considering the pathological value of inflammation, it is crucial that future studies investigate if a modulation of the systemic inflammatory reaction relieves severity of AS and opposes development of heart failure.

## 1. Introduction

Aortic valve stenosis (AS) is the third leading cardiovascular disease in the developed world [1] with increasing prevalence in the ageing population [1], and untreated AS of any degree causes premature mortality [2], which for a relevant amount of patients is caused by acute decompensation because of heart failure in acute advanced AS [3]. Due to the left ventricular outflow obstruction, which reduces cardiac output, patients with symptomatic AS suffer from decreased exertional tolerance, from exertional dyspnoea and dizziness, angina pectoris and syncope, and the disease may end in heart failure [4]. However, to date no therapy exists to prevent or heal the disease, except for surgical or catheter aortic valve replacement [4]. Today, it is accepted that AS, initially considered as purely degenerative, is an active process, comprising a local chronic inflammatory reaction and calcification [5], while systemic cytokine and immune cell levels were observed to have predictive value regarding long-term mortality after transcatheter aortic valve implantation (TAVI) [6]. However, little is known about the interplay between local processes in the stenotic aortic valve or the hypertrophic myocardium and the systemic immune system.

On valve level, biomechanical as well as metabolic processes stimulate valvular endothelial cells (VECs), which upregulate adhesion molecules, resulting in binding and invasion of circulating inflammatory cells into the valve tissue. The recruited as well as local immune cells in the aortic valve stroma secrete cytokines and thereby induce an extracellular matrix (ECM) remodelling and promote activation and calcification of valvular interstitial cells (VICs) [5]. Yet, to date, the effects of inflammatory cytokines and related factors on valve pathology upon AS have not been thoroughly studied.

Myocardial inflammation has recently emerged as a pathophysiologic process contributing to cardiac hypertrophy, fibrosis, and dysfunction upon stressors as, e.g., hemodynamic stress or pressure overload, if initially adaptive mechanisms become maladaptive upon persistence of the stressor. Those stressors directly induce the secretion of cytokines, chemokines, and adhesion molecules by cardiomyocytes, fibroblasts, and endothelial cells, promoting myocardial recruitment of monocytes, neutrophils, and lymphocytes [7,8,9,10,11]. Accumulated macrophages and lymphocytes secrete more cytokines and amplify the inflammatory reaction [9], and especially T cell recruitment to the myocardium is responsible for cardiac remodelling via TGF-β and Smad signalling [7].

Lymphocytes originate in lymphoid organs, undergo clonal expansion by proliferation after antigen presentation by antigen presenting cells (APCs), and thereafter emigrate from the lymphoid organs as primed T and B cells. In the body, via interaction with adhesion molecules on endothelial cells and adhesion-mediated chemokines [12], which are both highly increased under inflammatory conditions [13], the lymphocytes enter the parenchyma of peripheral organs, a process which is called “homing” [14]. To prevent autoimmunity, lymphocyte activation besides antigen presentation requires a secondary stimulus. These costimulatory signals [15,16,17,18] are presented by APCs and bind to their respective receptors on T and B cells, which are upregulated in the presence of inflammatory stimuli [15,18].

The murine wire injury (WI) model was previously shown to successfully induce AS, displaying characteristic features of the human disease, as increased blood flow velocity over the aortic valve, valve thickening, fibrosis, calcification, and a local inflammatory reaction [19].

The aim of this study is to find out if a systemic inflammatory reaction is present in the WI model, and if so to investigate the mechanisms linking local processes and the systemic inflammatory reaction, with a focus on the adaptive immune reaction in this model of a chronic disease.

For this purpose, mice were subjected to WI surgery to induce moderate AS or sham surgery, and four weeks after surgery, immune cell numbers, costimulatory molecule expression, and cytokine levels were investigated locally in the aortic valve and in the myocardium, as well as in the periphery (blood, lymph nodes, and spleen as secondary lymphoid organs, and liver). Echocardiography, conducted prior to sacrifice, served to assess successful induction of AS, as well as left ventricular function and structure, and was correlated with markers of a local and systemic inflammatory reaction to obtain descriptive information about mechanistic links between the inflammatory reaction, AS severity and markers of cardiac function and hypertrophy.

This study in the WI mouse model of AS found a local cardiac inflammatory reaction in the myocardium and aortic valve, and a systemic inflammatory reaction. These encompass changes in immune cell numbers and ratios, as well as levels of cytokine and costimulatory molecule expression on adaptive immune cells. Correlations of these parameters with AS severity, left ventricular function, and hypertrophy were observed. As novel finding of this study, these results hint at a systemic inflammatory reaction upon murine AS and its close mechanistic link with the local processes in the heart, including heart function.

## 2. Materials and Methods

### 2.1. Animals

For all experiments, 8–12-week-old male C57Bl/6J mice were used. Animal experiments were performed in accordance with the Directive 2010/63/EU of the European Parliament on the protection of animals used for scientific purposes and the national guidelines on animal care. They were approved by the Landesamt für Natur, Umwelt, und Verbraucherschutz (LANUV, Nordrhein-Westfalen, Germany) under file reference 84-02.04.2017.A172. All animals used in this study were purchased from Janvier Labs (Le Genest-Saint-Isle, France) and kept at the central facility for animal research and scientific animal welfare tasks (ZETT) of the Heinrich Heine University, Düsseldorf, Germany. They were fed with a standard chow diet, received tap water ad libitum and were maintained at 22 °C under a 12 h light/dark cycle on wood chips.

Mice were randomly divided into three groups (control, sham, or WI surgery) using unbiased assignment. To avoid confounders, sham and WI animals were housed in the same cages, number, and time points of interventions (surgery, application of analgesics, and echocardiographic investigations) and sacrifice were similar. The control group served to detect additional confounders, as sequelae of interventions, by sharing that they were housed in the same animal rooms, but they were not treated or transported prior to time point of sacrifice.

Power calculation was performed with G*Power (Version 3.1), using a significance level of 0.05, 80% power and two-sided *t*-test, based on previous experiences with this model. According to previous experience, group differences of 10% can be measured in groups of *n* = 8 animals. Mortality due to wire injury of the aortic valve is on average 10%, and about 25% of animals must be excluded as they present with exclusion criteria (please see below). These factors were considered upon determination of animal numbers (please see Table 1).

In total, group sizes for flow cytometry of activation markers and CD4/CD8 ratio were (9/7/8), for RT-qPCR (9/8/13), for multiplex immunoassay (4/9/8) and for PFC incubations as well as immune cell numbers in the blood (9/18). Numbers represent age-matched untreated control mice only used for organ collection, sham-operated, and animals after wire injury surgery (WI) to induce AS.

Echocardiography was performed in all sham and WI animals, but not in control animals of which the organs were collected, without subjecting mice to any additional procedure but killing. For all the other analyses, separate groups of operated animals were required as the small amount of available tissue from one animal could only be used for one analysis method.

For PFC incubation experiments, only animals after surgery (sham and WI) were investigated, as the required blood samples were collected from mice of which the tissues subsequently were subjected to investigations independent of this study. With the aim to reduce animal numbers in line with 3R and aiming at using as many tissues of sacrificed animals as possible; therefore, in this experiment, no additional control animals were killed, of which the remaining tissues would not have been of use.

Inclusion criteria were unaltered blood flow velocity over the aortic valve after surgery for the sham group, and an increase to at least 2000 mm/s or 1.5 of baseline for the WI group. Out of the whole *n* = 69 operated animals, animals which died during surgery (*n* = 12), or reached a humane endpoint (*n* = 1) prior to study endpoint four weeks after surgery, and one animal with severe aortic regurgitation showing a diastolic backward flow into the left ventricle were excluded from the analysis.

### 2.2. Echocardiography

For verification of AS induction, exclusion of development of aortic valve insufficiency (AI) and assessment of left ventricular function and structure, echocardiography was performed on a Fujifilm VisualSonics Vevo 3100 Ultra high frequency imaging platform with Vevo lab software (Version 2.2.0), and an MX550D transducer (Fujifilm VisualSonics, Toronto, ON, Canada). Data were recorded at baseline, one and four weeks after surgery. During the procedure, mice were anesthetized with 2% isoflurane under continuous monitoring of echocardiogram (ECG), respiratory rate, and body temperature. The chest of mice was depilated, and pre-warmed bubble-free ultrasound gel was applied to allow artefact-free image acquisition. Aortic valve peak velocity was measured in suprasternal view with a pulsed wave Doppler using angle correction between 45° and 55°. An increase in peak velocity to at least 2000 mm/s or 1.5 of baseline was considered as successful induction of moderate AS, as described previously [19].

To assess left ventricular function, left ventricular ejection fraction, fractional shortening, cardiac output, stroke volume, left ventricular volumes, cardiac mass and left ventricular wall thicknesses were measured in parasternal long-axis view using the Vevo LV-Trace function. AI was imaged using colour-Doppler mode in the left lateral view [19].

### 2.3. Wire Injury Surgery (WI) to Induce AS 

AS was induced in mice as described previously [20] after an acclimatization period of seven days subsequent to delivery to the animal facility from the breeder. In brief, mice were anesthetized by intraperitoneal injection with a mixture of ketamine (100 mg/kg body weight [BW]) and xylazine (10 mg/kg BW). After loss of posture reflexes, mice were placed in a supine position on a warming pad. The skin on the ventral neck was cut in midline, and the right carotid artery was exposed. After ultimate distal ligation with a 5-0 silk suture and temporal proximal ligation with a metal clip, a coronary guide wire (Abbott HI-TORQUE 0.014”; Abbott Cardiovascular, Plymouth, MN, USA), after careful opening of the metal clip, was inserted into the carotid artery, fixed loosely in the vessel with another silk suture to prevent bleeding, and advanced beyond the aortic valve level under echocardiographic guidance to prevent destroying the valve or harming the myocardium. Here, it was moved forwards and backwards 10 times and rotated 50 times as described before for induction of moderate AS [21]. After removal of the guide wire, the right carotid artery was ultimately ligated proximally with the 5-0 silk suture to prevent bleeding. For sham operation, an identical surgery was performed, but the wire was not passed over the aortic valve and rotations were performed above valve level.

For post-surgery analgesia, 0.05–0.1 mg/mL of buprenorphine in a maximum volume of 10 mL/kg body weight was applied subcutaneously every 6–8 h for 72 h following surgery. Scoring to assess pain or distress was performed once per day in the first three days after surgery, and once per week thereafter, with daily adspection of animals. Humane endpoints were weight loss of more than 20% or more than 15% within two days, signs of severe heart insufficiency (dyspnea, reduced well-being and movement, and weight loss), spasms, paralysis, signs of severe suffering (automutilation, aggression, squatting, and shivering), movement disorders (circling), and suture dehiscence.

### 2.4. Ex Vivo Analysis—Flow Cytometry, RT-qPCR, and Multiplex Immunoassay

Animals were sacrificed four weeks after WI by exsanguination in deep anesthesia with ketamine (100 mg/kg BW) and xylazine (10 mg/kg BW), injected intraperitoneally. Whole blood collection was carried out via transthoracic heart puncture with a pre-heparinized syringe. Thereafter, heart, regional and peripheral lymph nodes, spleen, and liver were excised and immediately washed with ice-cold phosphate-buffered saline (PBS, Carl Roth GmbH, Karlsruhe, Germany), followed by weighing of the heart, spleen, and liver on a precision balance.

### 2.5. Flow Cytometry for Assessment of Total Immune Cell Numbers and Costimulatory Molecule Expression on Immune Cells Isolated from the Heart and Peripheral Immune Organs

For isolation of immune cells from organs, aortic valves were dissected from hearts, and organs were cut into pieces and digested in a collagenase solution (0.3% collagenase II [SERVA, Heidelberg, Germany], 0.1% DNAse [Merck, Darmstadt, Germany] in PBS) for 15 min at 37 °C under permanent agitation. Thereafter, organs were ground through a 40 µm cell strainer (Merck) to obtain a single cell suspension.

For single cell suspensions obtained from organs as well as for whole blood, a red blood cell lysis (in-house prepared ammoniumchloride lysis buffer pH 7.4) was performed for 10 min on ice. After washing with 200 µL MACS buffer (0.5% BSA fraction V [Carl Roth GmbH], 5 mM EDTA [ethylendiaminetetraacetic acid, Carl Roth GmbH] in PBS pH 7.4), this was followed by a 5% FcR block (Miltenyi Biotec, Bergisch-Gladbach, Germany) in MACS buffer for 10 min on ice.

Immune cell numbers in the blood were estimated by counting total number of monocytes, neutrophils, and T and B cells in 10,000 events. To calculate the absolute number of immune cells per organ, the complete organ was dissociated and taken up in the same amount of MACS buffer for every experiment, from which a predefined volume (or all, in case of the aortic valve) was collected, in which all immune cells were counted. The total number of leukocytes, monocytes, neutrophils, lymphocytes, and T and B cells in the respective organ were then extrapolated from this part-volume. The rest of the cell suspension was evenly distributed, in a manner that all combinations of costimulatory markers on all relevant immune cell types could be measured in the respective organs of one animal.

After an additional washing step, cells were stained with CD45 (BD Biosciences, Heidelberg, Germany, clone 30-F11), CD11b (Biolegend, San Diego, CA, USA, clone M1/70), Ly6G (Biolegend, clone 1A8), CD3 (Biolegend, clone 145-2C11), CD19 (BD Biosciences, clone 1D3), MHCII (Biolegend, clone M5/114.15.2), CD11c (ThermoFisher Scientific, Waltham, MA, USA, clone N418) and CD8a (Biolegend, clone 53-6.7) to discriminate total immune cells (CD45^+^), myeloid cells (CD45^+^ + CD11b^+^), lymphoid cells (CD45^+^ + CD11b^−^), neutrophils (CD45^+^ + CD11b^+^ + Ly6G^+^), monocytes (CD45^+^ + CD11b^+^ + Ly6G^−^), T cells (CD45^+^ + CD11b^−^ + CD3^+^), B cells (CD45^+^ + CD11b^−^ + CD19^+^), DCs (dendritic cells, CD45^+^ + CD11c^+^ + MHCII^+^), CD8a, and CD11b DCs. As costimulatory molecules, CD40 (Biolegend, clone 3/23), CD40L (ThermoFisher Scientific, clone MR1), CD62L (Biolegend, clone MEL-14), CD69 (Biolegend, clone H1.2F3), CD70 (Biolegend, clone FR70), CD80 (ThermoFisher Scientific, clone 16-10A1) and CD86 (BD Biosciences, clone GL1) were assessed. CD4 (Biolegend, clone RM4-5) and CD8a (BD Biosciences, clone 53-6.7) were used to assess the CD4/CD8 ratio. Staining with primary labelled antibodies was performed for 30 min in the dark on ice. After an additional washing step, cell pellets were taken up in 200 µL MACS buffer containing 1 µg/mL 4′, 6-Diamidino-2-phenylindole (DAPI, Sigma-Aldrich, München, Germany). Flow cytometry to assess mean fluorescence intensity (MFI) of costimulatory molecules on the respective immune cell subtype and for the calculation of the CD4/CD8 ratio was performed on a FACS CantoII, run with FACS Diva software (Version 8.0.3, BD Biosciences). Data analysis was performed with FlowJo (Version 7.6, BD Biosciences).

### 2.6. RT-qPCR

For RT-qPCR, aortic valves were prepared from the heart and organs were cut into pieces and immersed in a 10-fold volume of RNAlater (ThermoFisher Scientific) at room temperature for 25–72 h before storage at −80 °C.

T cells were isolated from whole blood after red blood cell lysis with the Pan T cell isolation kit mouse (Miltenyi Biotec) according to manufacturer’s instructions and stored as a pellet at −80 °C.

For RNA extraction, tissue lysates or cells, respectively, were thawed on ice and thereafter minced in RLT buffer with a Retsch TissueLyser II Bead Mill Sample Disrupter (Qiagen, Hilden, Germany). RNA was extracted with the RNeasy Micro Kit (Qiagen) following manufacturer’s instructions.

cDNA transcription was performed with the SuperScript™ VILO™ cDNA-Synthesis Kit (ThermoFisher Scientific) according to manufacturer’s instructions.

For aortic valves, regional and peripheral lymph nodes, thereafter, a preamplification of genes of interest was performed using the TaqMan™ PreAmp Master Mix Kit (ThermoFisher Scientific) following manufacturer’s instructions.

RT-qPCR was performed on a QuantStudio 7 Flex Real-Time-PCR System (ThermoFisher Scientific) with TaqMan probes against *IL-6* (Mm00446190_m1), *TNF-α* (Mm00443258_m1), *IL-10* (Mm00439614_m1), *CD69* (Mm01183378_m1), *Foxp3* (Mm00475162_m1), *CD44* (Mm01277163_m1), *CD3* (Mm01179194_m1), *CD4* (Mm00442754_m1), *CD40* (Mm00441891_m1) and *CD40L* (Mm00441911_m1).

Relative gene expression in relation to the housekeeping gene *GAPDH* (Mm99999915_g1) was calculated using the Delta-Delta Ct Method.

### 2.7. Cytokine Quantification with Multiplex Immunoassay

To obtain plasma, whole blood was centrifuged for 15 min at 10,000× *g* at room temperature. Plasma was stored at −80 °C.

After explantation of aortic valves, the myocardium was cut into pieces and proteins were extracted from the myocardium using the Bio-Plex^®^ Cell Lysis Kit (Bio-Rad Laboratories, Hercules, CA, USA) according to manufacturer’s instructions, performing tissue homogenization with a Retsch TissueLyser II Bead Mill Sample Disrupter (Qiagen). The protein extract was stored at −80 °C.

Protein amounts in the plasma and cell lysate were assessed with the DC Protein assay (Bio-Rad Laboratories) immediately, prior to the cytokine measurements.

Quantification of cytokines in the samples was performed with the Bio-Plex Pro Mouse Th17 Immunoassay (Bio-Rad Laboratories), using a Bio-Plex Pro Wash Station and a Bio-Plex 200 system (Bio-Rad Laboratories) according to manufacturer’s instructions.

### 2.8. Synthesis of Perfluorocarbon Nanoemulsions (PFCs)

PFC nanoparticles were produced as described in another study [22]. In brief, 4% (*w*/*w*) phospholipid (Lipoid E80S, Lipoid GmbH, Ludwigshafen, Germany) was dissolved in 10 mM phosphate buffer (7 mM Na_2_HPO_4_ [Carl Roth GmbH], 3 mM NaH_2_PO_4_ [Carl Roth GmbH], pH 7.4 isotonized with 2.5% (*w*/*w*) glycerol [Carl Roth GmbH]) and pre-emulsified for 30 min on a stirrer. Afterwards, 10% (*w*/*w*) perfluoro-15-crown-5 ether [Fluorochem Ltd., Hadfield, UK] and 0.0025 mol% Cy5-labelled phosphatidylethanolamine (Avanti Polar Lipids Inc., Alabaster, AL, USA) were added, followed by a 30 min period of stirring in the dark. Thereafter, a crude emulsion was formed by high shear mixing (Ultra Turrax TP 18/10, IKA-Werke GmbH & Co. KG, Staufen, Germany). The final emulsion was produced by high shear homogenization over 10 cycles at 1000 bar, using an M-110P Microfluidizer (Microfluidics International Corporation, Westwood, CA, USA). PFCs were heat-sterilized in glass vials (VWR, Radnor, PA, USA) under standard conditions (121 °C, 1 bar, 22 min).

### 2.9. PFC Incubations of Blood Immune Cells

Immune cells were isolated from whole blood as described above, followed by an FcR block. Thereafter, cells were resuspended in DMEM (Dulbecco´s modified eagle´s medium, Merck), and after collecting one baseline sample, 5 µL Cy5-PFCs were added to 1 mL of cell suspension. After 5, 10, 20, 40, and 80 min each, a 100 µL sample was collected, transferred into 2 mL cold MACS buffer, and stored on ice. When all samples had been collected, after one washing step with MACS buffer, antibody staining was performed using CD45 (BD Biosciences, clone 30-F11), CD11b (Biolegend, clone M1/70), Ly6G (Biolegend, clone 1A8), CD3 (Biolegend, clone 145-2C11) and CD19 (BD Biosciences, clone 1D3) to discriminate monocytes, neutrophils, and T and B cells. After one centrifugation step, cell pellets were taken up in 200 µL MACS buffer with 1 µg/mL DAPI.

Flow cytometry to assess the percentage of positive cells of every immune cell subtype was performed on a FACS CantoII, ran with FACS Diva software (Version 8.0.3, BD Biosciences). Data analysis was performed with FlowJo (Version 7.6, BD Biosciences).

### 2.10. Statistical Analysis and Blinding

For statistical analysis, Graph Pad Prism (Version 8.0, GraphPad Software, Boston, MA, USA) was used. Due to small sample sizes of *n* < 10 per group not allowing for reliable normality testing, with normal distribution as prerequisite for using parametric tests with sufficient reliability, non-parametric tests were performed [23]. To compare two groups the Mann–Whitney U test was used, to compare three groups the Kruskall–Wallis test with Dunn’s multiple comparison post hoc test was used. As Prism does not offer a non-parametric alternative, to compare changes over time for two different treatments, two-way ANOVA with Bonferroni post hoc test was applied.

Separate correlation analyses between different parameters of inflammation and AS severity, as well as left ventricular function and structure, were performed with the Spearman correlation analysis.

Both in the Mann–Whitney U and Kruskall–Wallis tests, and Spearman correlation analyses, only the control, sham, and AS groups for one, or two variables, respectively, were compared. This implies that no multiple comparisons were performed and no adjustment for multiple comparisons were required to prevent type I errors.

Two-sided tests were used for all statistical tests. Data shown are mean ± SD and *p*-values of 0.05 or less were considered statistically significant, and values of 0.5 or less as trends. The approach of pointing out trends was chosen to not overlook potentially biologically relevant effects when using the conservative non-parametric tests, while at the same time emphasizing that those should not be overinterpreted [24]. R-values larger than 0.5 or smaller than −0.5 were considered as signs of a fair correlation according with handling of correlation coefficients in the literature [25]. Data analysis was performed by a blinded investigator.

## 3. Results

### 3.1. WI Successfully Induces AS Development and Impacts on Heart Function and Structure

**Figure 1:**
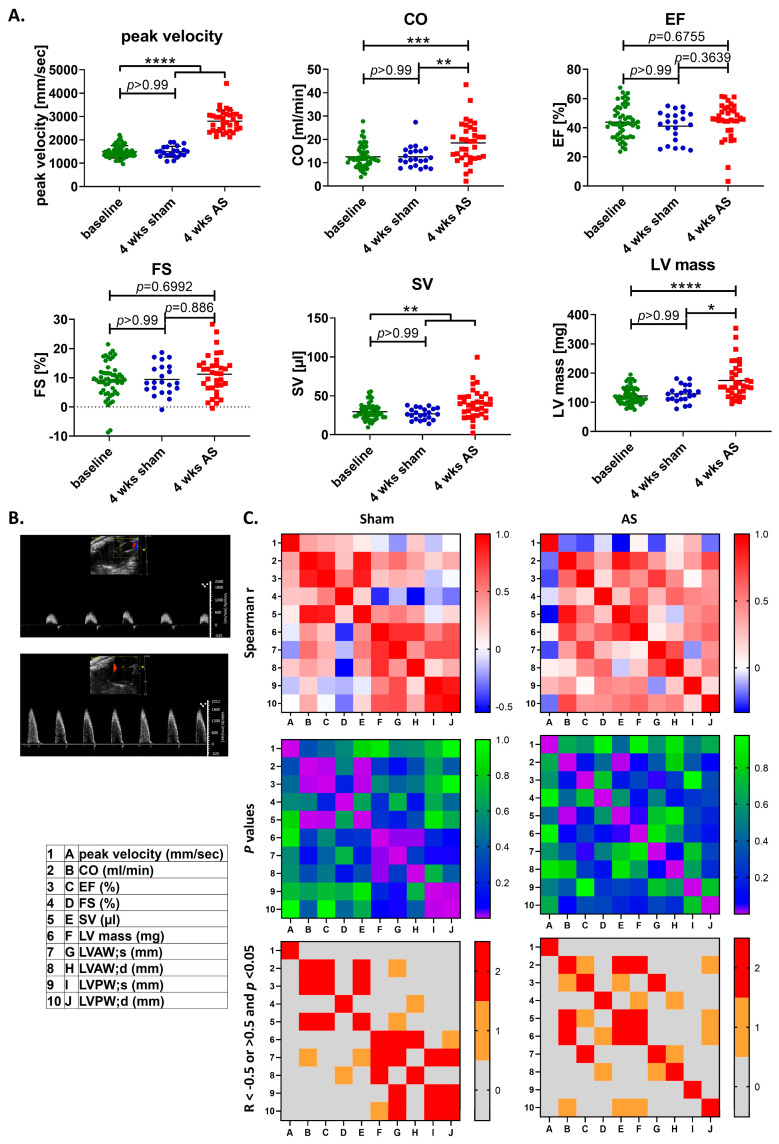
Assessment of the AS development, heart function, and structure with echocardiography. Echocardiography at baseline, one and four weeks after WI was used to assess severity of AS and left ventricular function and structure. (**A**) Peak blood flow velocity, cardiac output, ejection fraction, fractional shortening, stroke volume, and left ventricular mass were compared between baseline and animals four weeks after sham or WI surgery using the Kruskall–Wallis test with Dunn’s multiple comparison post hoc test. (**B**) Exemplary images of echo loops at baseline (upper) and after successful induction of AS (lower). (**C**) Spearman correlation analysis between echocardiographic parameters was performed for WI animals four weeks after surgery. Upper: correlation matrices showing Spearman’s R; middle: matrices showing *p*-values of correlation analysis; and lower: matrices showing values with R smaller or larger than 0.5 (first criterion) and *p*-values lower than 0.05 (second criterion) in red, with only one criterion in orange, and no criterion in grey. AS: aortic valve stenosis, wks: weeks. Data in (**A**) are mean values ± SD. CO: cardiac output, EF: ejection fraction, FS: fractional shortening, LV: left ventricle, LVAW;d: end-diastolic left ventricular anterior wall; LVAW;s: end-systolic left ventricular anterior wall, LVPW;d: end-diastolic left ventricular posterior wall, LVPW;s: end-systolic left-anterior posterior wall, SV: stroke volume. (**A**,**C**) display values of *n* = 9 (sham) and *n* = 8 (WI) animals; * = *p* < 0.5, ** = *p* < 0.01, *** = *p* < 0.001, **** = *p* < 0.0001.

In the first step, assessing echocardiographic parameters of left ventricular function and hypertrophy, and performing a correlation analysis between the different parameters four weeks after AS, left ventricular mass in this stage of AS was found to be positively correlated with cardiac output and stroke volume, and trended toward positive correlation with fractional shortening (Figure 1A,C).

This indicates compensatory mechanism ensuring preserved heart function upon mild cardiac hypertrophy in early AS.

### 3.2. Heart and Immune Organ Weights and Immune Cell Numbers Trend to Correlate with Echocardiographic Parameters upon AS

**Figure 2:**
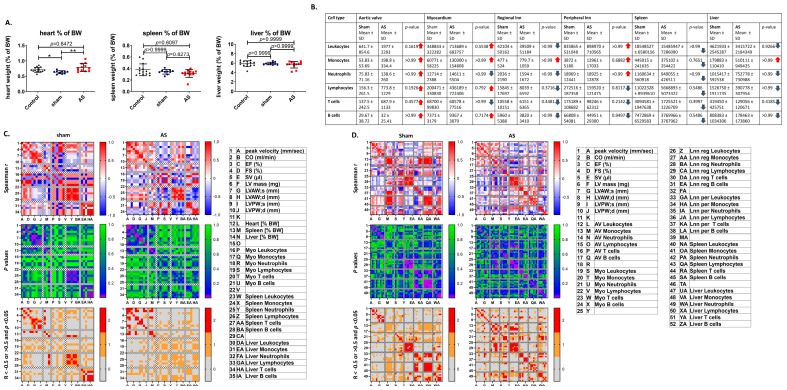
Organ weight and immune cell numbers in the heart and peripheral immune organs upon AS. Body weight, explanted heart, spleen, and liver weight of mice were determined four weeks after surgery, and total number of immune cells per organ was measured with flow cytometry. (**A**) Percentage of heart (**left**), spleen (**middle**), and liver (**right**) of the total body weight of control animals and mice four weeks after sham or WI surgery was compared using Kruskall–Wallis test with Dunn’s multiple comparison post hoc test. Red arrows: mean of AS higher than of sham, blue arrows: mean of AS lower than of sham. Data are mean values ± SD. (**B**) Analysis of differences in leukocyte numbers in different organs between sham and AS animals using the Mann–Whitney test. (**C**) Spearman correlation analysis between organ weights of heart, spleen, and liver, leukocyte numbers in the respective organs, and echocardiographic parameters. (**D**) Spearman correlation analysis between leukocyte numbers in organs (aortic valve, myocardium, regional and peripheral lymph nodes, spleen, and liver) and echocardiographic parameters. Upper: correlation matrices showing spearman R; middle: matrices showing *p*-values of correlation analysis; and lower: matrices showing values with R smaller or larger than 0.5 (first criterion), and *p*-values lower than 0.05 (second criterion) in red, with only one criterion in orange, and no criterion in grey. AV: aortic valve, BW: body weight, Lnn per: peripheral lymph nodes, Lnn reg: regional lymph nodes, Myo: myocardium. Shown are data of *n* = 9 (control), *n* = 7 (sham), and *n* = 8 (WI) animals; * = *p* < 0.5, ** = *p* < 0.01.

**Figure A2:**
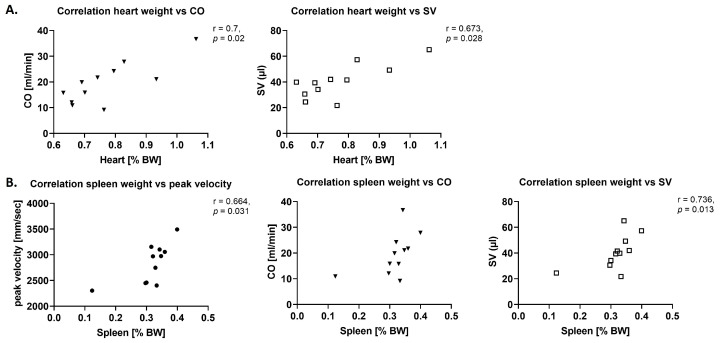
Organ weight in the heart and peripheral immune organs upon AS. Body weight, explanted (**A**) heart, or (**B**) spleen, and liver weight of mice were determined four weeks after surgery. The Spearman correlation analysis between organ weights and echocardiographic parameters was performed for WI animals. Displayed are graphs of significantly correlated parameters. Data are values of *n* = 15 WI animals; *p* < 0.05 was considered statistically significant.

To investigate if systemic effects are present in this AS model, in a first experiment, while heart weight was found to be increased (sham: 0.6269 ± 0.0486%, WI 0.7809 ± 0.131%, *p* = 0.0013), weights of spleen and liver were unaltered in WI (Figure 2A). Correlation analysis between organ weights and echocardiographic parameters revealed positive r-values up to significant correlations of weight with AS severity, heart function and echocardiographic hypertrophy markers, while in sham animals, r-values for heart function or hypertrophy markers were negative with significance of several parameters for spleen weight (Figure 2C and Figure A2).

These observations support the hypothesis that there may be an association between weight of the secondary immune organ spleen and heart function and hypertrophy in AS, suggesting a systemic, probably inflammatory reaction.

To further pursue this hypothesis, the study in the next step counts immune cell numbers in heart, peripheral immune organs, and liver by flow cytometry of dissociated organs. A trend towards increased numbers of T cells in the aortic valves, unchanged leukocyte numbers in the myocardium, and a trend towards reduced numbers of T cells in regional and peripheral lymph nodes, spleen, and liver were revealed (Figure 2B).

While spleen leukocyte numbers showed negative r-values in both WI and sham, liver leukocyte numbers displayed positive r-values upon correlation with heart weight upon WI or negative r-values for sham (excluding a positive r-value for neutrophils in sham). These observations suggest that cardiac hypertrophy in WI is accompanied by systemic inflammatory reactions, going in hand with T cell recruitment from the peripheral immune organs to the aortic valve. In the liver, T cell loss seems to be oppositely regulated with a less severe recruitment in more severe cases of cardiac hypertrophy, which is in line with reported observations of immune cell retention in the liver in the presence of, e.g., increased peripheral IL-6 levels upon myocardial infarction (MI) [26].

Myeloid cell numbers in the myocardium showed positive r-values for correlation with spleen and liver weight in WI (sham: monocyte numbers positive r-values for spleen, negative r-values for liver weight, and opposingly for neutrophil numbers) hypothetically due to swelling of those organs parallel to the acute component of the inflammatory reaction in the heart. In contrast to this, r-values for myocardial lymphocyte numbers were negative for correlation with spleen and liver weight upon WI and sham, supporting the hypothesis of lymphocyte recruitment from those organs to the heart, as was similarly reported for the spleen upon MI as important leukocyte storage pool [27] (Figure 2C).

A significant positive correlation between B cell numbers in the aortic valve and left ventricular mass was observed in WI, while it trended towards significance in sham, which is so far in line with previous reports, as those describe B cells in the aortic valve upon AS to be correlated with severity of the stenosis [28].

Myocardial B cell numbers trended towards a negative correlation with cardiac output, ejection fraction and stroke volume in WI (positive r-values in sham), in line with the literature on MI [29].

R-values of regional lymph node leukocyte numbers were mildly positive for the correlation with AS severity and mainly negative for the correlation with heart function and hypertrophy parameters both in WI and sham, supporting the hypothesis of recruitment of immune cells from this compartment to the heart upon AS, and a connection with preserved cardiac performance parallel to myocardial hypertrophy. In addition, correlations were significantly positive for regional lymph node myeloid cells and aortic valve neutrophils, and significantly negative for all immune cell types with aortic valve T cells, while no such pattern was observed in sham, strengthening the hypothesis of T cell recruitment to the aortic valve, while the acute component of the aortic valve inflammation rather seems to go in hand with a systemic inflammatory reaction.

For peripheral lymph nodes and spleen, leukocyte r-values trended to, respectively, be positive and negative for AS severity suggesting systemic inflammation and immune cell recruitment from the spleen, to be mainly negative for spleen leukocyte numbers and cardiac parameters of heart function and hypertrophy (sham: mainly positive), supporting immune cell recruitment from the spleen linked with better cardiac performance upon cardiac hypertrophy. In sham, r-values were uniquely positive for correlations between peripheral lymph node lymphocytes or spleen immune cells and aortic valve leukocytes (WI: heterogeneous), suggesting a mild systemic inflammatory reaction caused by the shear stress over the aortic valve already under steady-state conditions, as the aortic valve should not be harmed in sham surgeries, although this seems not to be accompanied by T cell recruitment.

**Figure 3:**
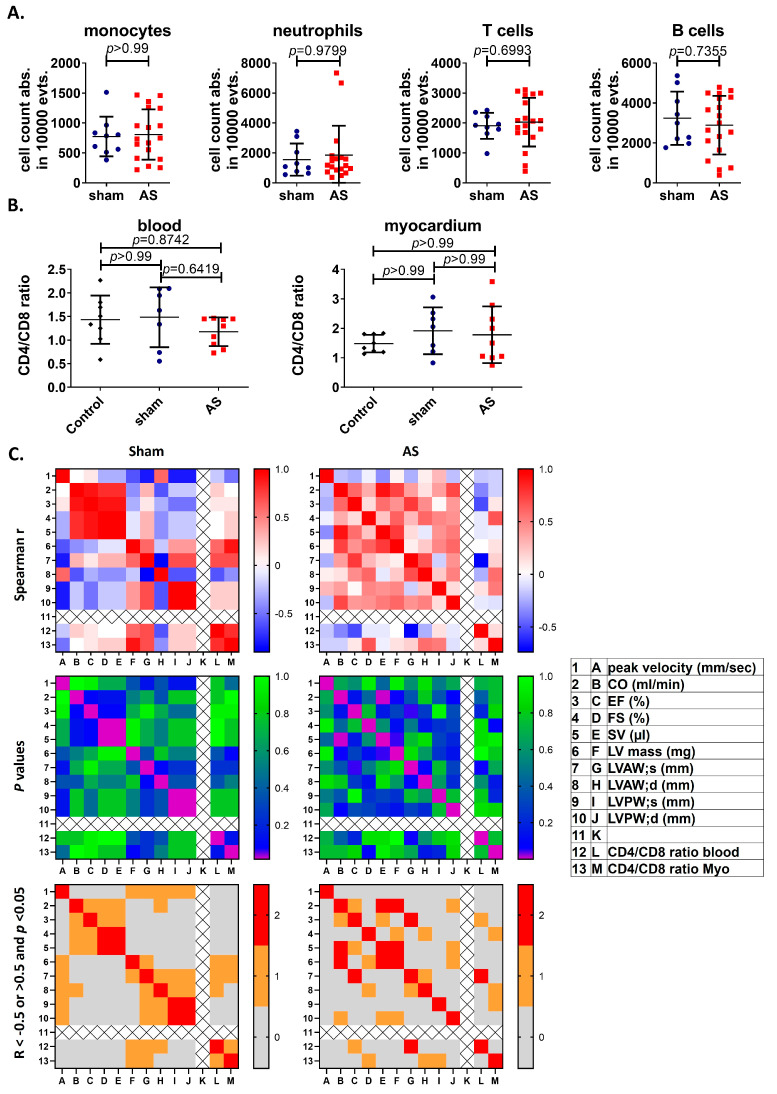
Immune cell numbers and the CD4/CD8 ratio in blood and myocardium upon AS. Flow cytometry of immune cells isolated from whole blood was performed. WI and sham groups were compared using the Mann–Whitney test (**A**). The CD4/CD8 ratio in the peripheral blood and myocardium was calculated and analyzed with Kruskall–Wallis test with Dunn’s multiple comparison post hoc test (**B**), and (**C**) a Spearman correlation analysis was performed between the CD4/CD8 ratio in blood or myocardium and echocardiographic parameters for WI animals. Upper: correlation matrices showing spearman R; middle: matrices showing *p*-values of correlation analysis; and lower: matrices showing values with R smaller or larger than 0.5 (first criterion) and *p*-values lower than 0.05 (second criterion) in red, with only one criterion in orange, and no criterion in grey. Data in (**A**) are mean values ± SD of *n* = 9 (sham) and *n* = 18 (WI) animals, in (**B**) mean values ± SD of (**B**,**C**) *n* = 9 (control), *n* = 7 (sham) and *n* = 8 (WI) animals.

Liver neutrophil numbers were up to significantly negatively correlated with cardiac wall thickness in WI (sham: positive r-values), suggesting a link between the peripheral acute inflammatory reaction and a less severe cardiac hypertrophy, which is in line with up to significant negative correlations of liver myeloid cell numbers with aortic valve T cell numbers as a sign of more severe AS. Additionally, a significantly positive correlation of liver B cell numbers, which as antigen-presenting and cytokine secreting cells are known activators and sometimes even stimulators of proliferation of T cells [30], with myocardial T cell numbers was found, while the correlation was not significant for sham (Figure 2D and Figure 3A).

### 3.3. CD4/CD8 Ratio in the Peripheral Blood Trends to Be Negatively, and in the Myocardium, to Be Positively Correlated with Heart Function upon AS

To further characterize the systemic inflammatory reaction, this study analyzed immune cell numbers in the blood four weeks after WI or sham surgery, observing no differences between WI and sham (Figure 3A). Also, calculating the blood CD4/CD8 ratio in the peripheral blood yielded no differences between sham and WI animals (1.484 ± 0.635 vs. 1.179 ± 0.3045, *p* = 0.6419) (Figure 3B). Correlation analysis of CD4/CD8 ratio with echocardiographic parameters; however, revealed negative r-values, which reached significance for anterior wall thickness, while in sham, r-values were mainly positive (Figure 3C), suggesting that CD4^+^ T cell levels in the blood upon WI may be related with reduced cardiac performance.

An increase in myocardial CD4^+^ T cell numbers is associated with an accelerated cardiac decompensation [31]; therefore, as a next step, the CD4/CD8 ratio in the myocardium was calculated, but no difference was found between sham and WI (Figure 3B). A correlation analysis of CD4/CD8 ratio with echocardiographic parameters even revealed a trend towards a positive correlation with fractional shortening, representing systolic cardiac function [32], and a trend towards a positive correlation with wall thickness parameters. This suggests an association of myocardial CD4/CD8 ratio with the mild hypertrophy serving heart function compensation at this early stage of AS (Figure 3C).

### 3.4. Cytokines, T Cell Activation Markers, and Costimulatory Molecules on mRNA Level Trend Towards Upregulation in Heart and Regional Lymph Nodes, but Towards Reduction in Peripheral Immune Organs upon AS

Based on the hypothesis, that upon WI, the mildly inflamed heart tissue induces a systemic immune reaction, while activated immune cells egress from peripheral immune organs to invade the aortic valve, in a next step, the study analyzed markers of immune cell activation. To this end, cytokine expression, early and chronic activation markers of T cells, and costimulatory molecule expression in heart and peripheral immune organs were determined on mRNA and protein level. Cytokines and co-stimulatory molecules were analyzed, as they serve to recruit and activate immune cells prior to clonal expansion and homing, while the focus on T cells arises from the chronic nature of the disease.

In the first step, the study investigated if transcription of cytokines, markers for early and chronic activation of T cells, and the costimulatory molecules *CD40* and *CD40L* [33], was altered in the aortic valve, myocardium, isolated peripheral blood T cells, and peripheral immune organs.

**Figure 4:**
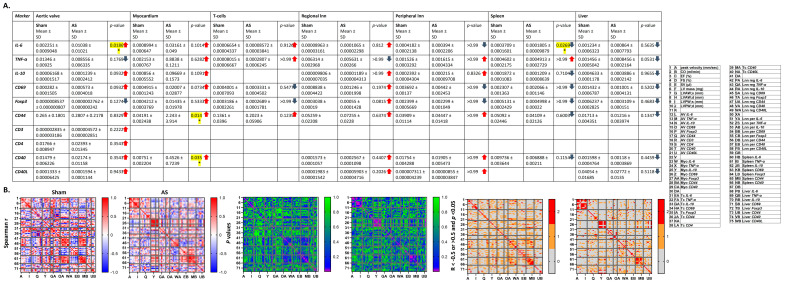
RT-qPCR analysis of cytokines, T cell activation markers, and costimulatory molecules upon AS. RT-qPCR was performed on isolated aortic valves, myocardium, T cells isolated from whole blood, and peripheral immune organs to assess cytokine levels, early and chronic activation markers of T cells and costimulatory molecules. (**A**) The Mann–Whitney test was used to assess differences between sham and WI animals for each mRNA and organ. Given are mean values ± SD and *p*-values, highlighted in yellow are significant *p*-values. (**B**) Spearman correlation analysis between mRNA levels and echocardiographic parameters was performed. Upper: correlation matrices showing spearman R; middle: matrices showing *p*-values of correlation analysis; and lower: matrices showing values with R smaller or larger than 0.5 (first criterion) and *p*-values lower than 0.05 (second criterion) in red, with only one criterion in orange, and no criterion in grey. Tc: T cells. Date comprise *n* = 9 (control), *n* = 8 (sham) and *n* = 13 (WI) animals, * = *p* < 0.05.

This analysis revealed a trend towards an increase in most cytokines, T cell activation markers and costimulatory molecules in aortic valve, myocardium, and regional lymph nodes, suggesting an inflammatory state, which should be especially marked in single T cells in the regional lymph nodes, as in those, T cell numbers according to the above-described results were reduced. The same markers in contrast, mostly trended towards reduction in spleen and liver after WI, in line with the reduced T cell numbers in these organs and supporting the hypothesis that there is some kind of systemic inflammatory reaction going on which also affects these tissues (Figure 4A).

Correlation analysis showed a mostly homogeneous picture of r-values, with a lower number of trends (r ≤ or ≥0.5) and significant values in WI compared with sham, with heterogeneity suggesting forces pushing inflammation marker expression in a certain direction, and homogeneity, therefore hinting at a loss of ordered conditions. R-values in aortic valves were mainly positive for the correlation between echocardiographic parameters and the different inflammation markers, except of mostly negative r-values for early T cell activation marker *Foxp3* and chronic T cell activation marker *CD44*, while most r-values were negative in sham, suggesting the inflammatory reaction in the aortic valve upon WI, known to go in hand with increased fibrosis [5] to be associated with more severe AS, but also with myocardial hypertrophy and preserved heart function.

Mostly positive r-values of cell activation markers in the spleen were found in correlation with echocardiographic hypertrophy markers (negative in sham).

Another interesting finding were the mainly negative correlations between regional lymph nodes or liver and inflammation markers measured in aortic valves, which is in line with the reduced T cell numbers in these organs in favour of those in the aortic valve as described above.

**Figure A4:**
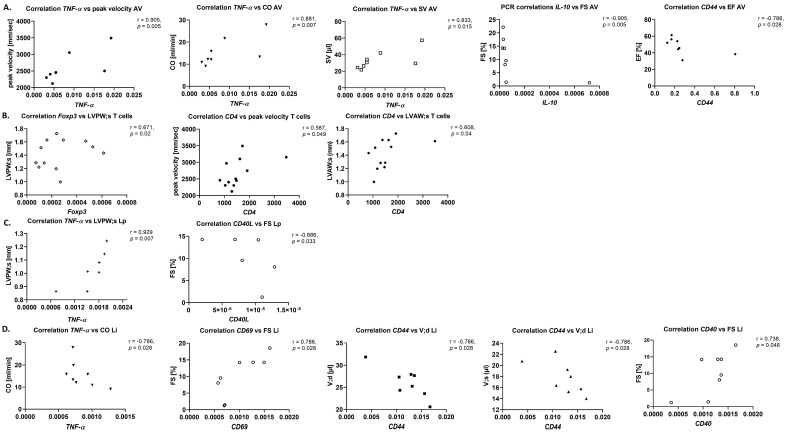
RT-qPCR analysis of cytokines, T cell activation markers and costimulatory molecules in heart, blood T cells and peripheral immune organs upon AS. RT-qPCR was performed on isolated (**A**) aortic valves, myocardium, (**B**) T cells isolated from whole blood and peripheral immune organs ((**C**) peripheral lymph nodes, (**D**) liver) to assess cytokine levels, early and chronic activation markers of T cells and costimulatory molecules. The Spearman correlation analysis between mRNA levels and echocardiographic parameters was performed for WI animals. Displayed are graphs of significantly correlated parameters. Data are values.

For the spleen, correlation with most inflammation markers in myocardium showed positive r-values up to significance in both WI and sham in line with the above-described positive r-values for correlation with cardiac hypertrophy, while most r-values were negative up to significance for the liver in WI but positive in sham animals, supporting the notion of a peripheral immune reaction upon myocardial inflammation (Figure 4B and Figure A4).

### 3.5. Cytokines on Protein Level in Blood Plasma and Myocardium Mildly Trend Towards Correlations with Echocardiography Parameters upon AS

**Figure 5:**
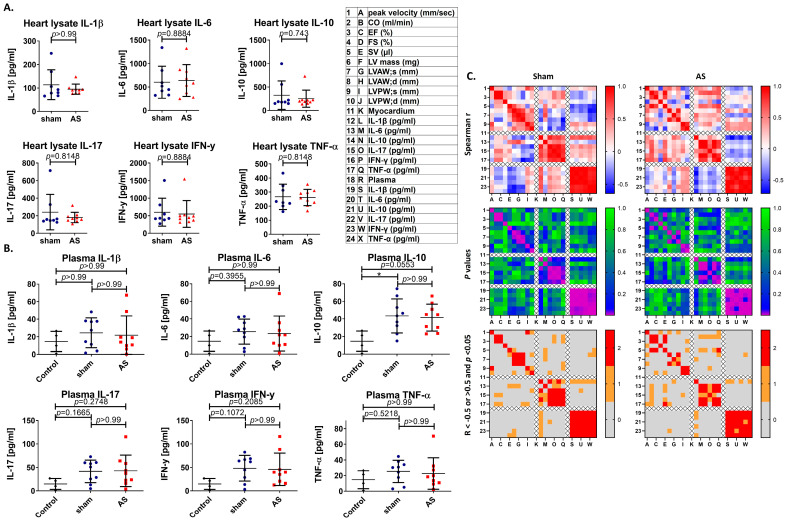
Multiplex immunoassay analysis of cytokines in blood plasma and myocardium upon AS. Multiplex Immunoassay was performed on blood plasma (**A**) and protein extract from myocardium (**B**) to assess Th17-cytokine levels. Analysis was performed with the Kruskall–Wallis test with Dunn’s multiple comparison post hoc test (**A**) or the Mann–Whitney test (**B**) to assess differences between control (**A**), sham and WI animals (**A**,**B**). (**C**) The Spearman correlation analysis between protein levels and echocardiographic parameters was performed. Upper: correlation matrices showing spearman R; middle: matrices showing *p*-values of correlation analysis; and lower: matrices showing values with R smaller or larger than 0.5 (first criterion) and *p*-values lower than 0.05 (second criterion) in red, with only one criterion in orange, and no criterion in grey. Data in (**A**,**B**) are mean values ± SD. Analyzed were n = 4 (control), n = 9 (sham) and n = 8 (WI) animals, * = *p* < 0.05.

**Figure A5:**
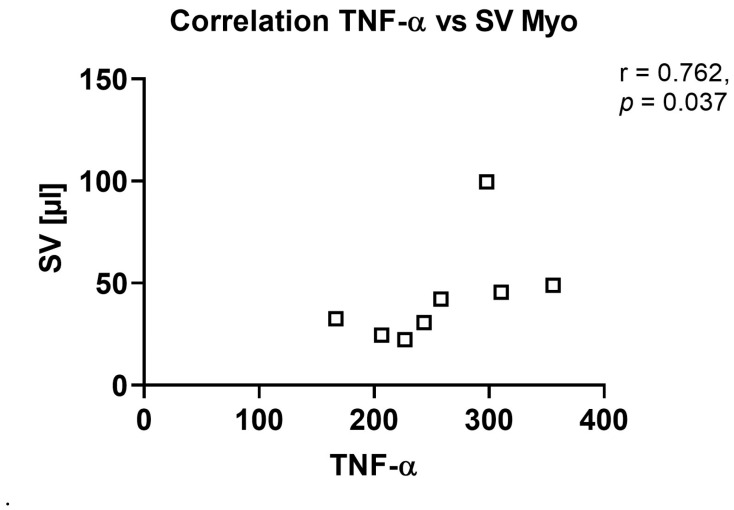
Multiplex Immunoassay analysis of cytokines in blood plasma and myocardium upon AS. Multiplex Immunoassay was performed on blood plasma and protein extract from myocardium to assess Th17-cytokine levels. Spearman correlation analysis between protein levels and echocardiographic parameters was performed for WI animals. Displayed are graphs of significantly correlated parameters. Data are values of *n* = 8 (WI) animals; *p* < 0.05 was considered statistically significant.

In the next step, this study investigates if cytokines in plasma and myocardial tissue were also altered on protein level upon WI. However, no differences between sham and WI animals were observed, while all markers in plasma trended towards an increase in mice after sham or WI surgery, compared with untreated control mice, suggesting inflammatory processes induced by the surgical intervention itself, independent of the presence of AS (Figure 5A,B and Figure A5).

A small number of correlations with echocardiographic heart function or hypertrophy markers, or between plasma and myocardial cytokine levels, was observed in both sham and WI animals. R-values were mostly positive (cardiac mass and left ventricular wall thickness) or unchanged, except of negative r-values for AS severity, stroke volume and anterior wall thickness for plasma cytokines in WI suggesting mainly a link between blood cytokine levels and cardiac hypertrophy (sham: negative except of positive r-values for peak blood flow velocity and posterior wall thickness). The positive r-values for peak blood flow velocity in sham, correspond with the above-described positive correlations of immune cell numbers in peripheral lymph nodes or spleen with immune cell numbers in the aortic valve in sham animals, suggesting a systemic immune reaction associated with shear stress over the aortic valve already in sham animals which in the case of plasma cytokines, in contrast to spleen immune cell numbers seems to be even related with a reduced cardiac performance (Figure 5C and Figure A5).

### 3.6. Costimulatory Molecule Correlation Pattern with Echo in Heart and Immune Organs Is More Homogeneous After WI Compared with Sham, While Cellular Uptake of Cy5-PFCs by T and B Cells Is Reduced upon AS

**Figure 6:**
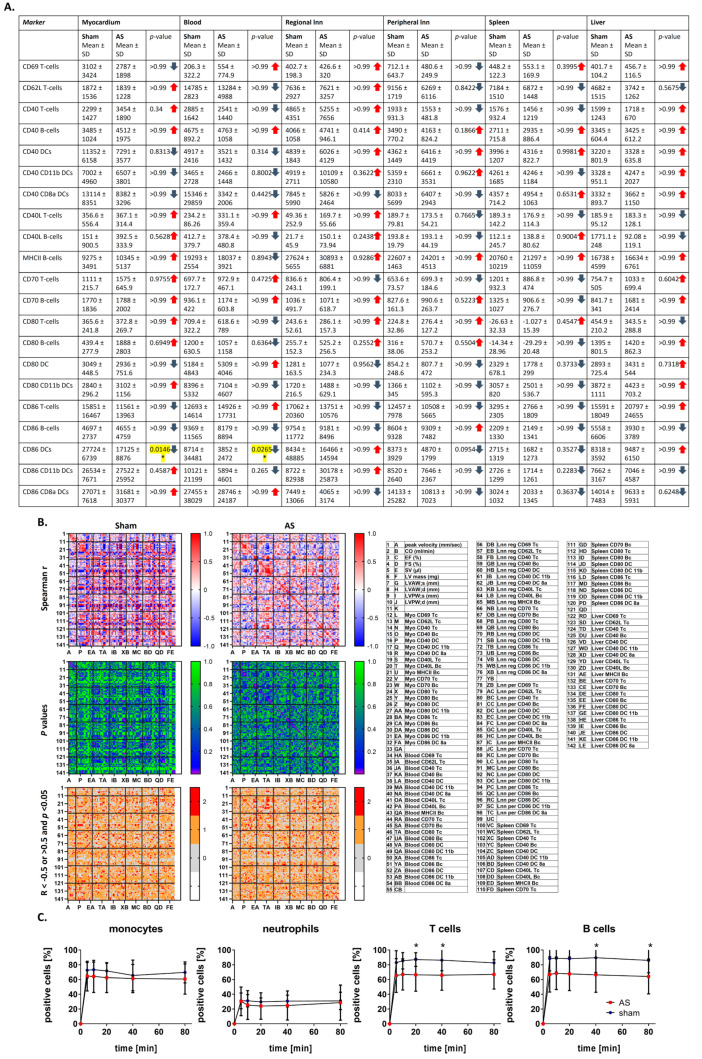
Costimulatory marker expression and cellular uptake of Cy5-PFCs upon AS assessed with flow cytometry. On T cells, B cells and DCs isolated from blood, heart or peripheral immune organs, expression of costimulatory markers was measured with flow cytometry. (**A**) The Mann–Whitney test was used to assess differences between sham and WI animals. Given are mean values ± SD and *p*-values, highlighted in yellow are significant *p*-values. (**B**) The Spearman correlation analysis between costimulatory molecules on the respective immune cell type, and echocardiographic parameters was performed. Upper: correlation matrices showing spearman R; middle: matrices showing *p*-values of correlation analysis; and lower: matrices showing values with R smaller or larger than 0.5 (first criterion) and *p*-values lower than 0.05 (second criterion) in red, with only one criterion in orange, and no criterion in grey. Data were collected from *n* = 9 (control), *n* = 7 (sham) and *n* = 8 (WI) animals, * = *p* < 0.05. (**C**) Immune cells isolated from blood were incubated with Cy5-PFCs, and percentage of positive immune cells over time was assessed with flow cytometry. Analysis was performed with two-way ANOVA with Bonferroni post hoc test. DC: dendritic cells. Data are mean values ± SD of *n* = 9 (sham) and *n* = 18 (WI) animals, * = *p* < 0.05.

In the next step, for a more detailed investigation on markers of immune cell activation, costimulatory molecule expression on T and B cells as well as on dendritic cells (DCs) in myocardium, blood and peripheral immune organs was analyzed using flow cytometry, revealing differential up- and down-regulation of costimulatory molecules (Figure 6A). Correlation analysis showed a more homogeneous pattern in WI compared with sham; however, the overall number of correlations was about similar between the two groups. For echocardiographic parameters, correlation analysis with the myocardium, blood, and peripheral organs for most costimulatory molecules yielded many trends towards and some significantly positive or negative (CD80 and CD86, liver only CD80) correlations with left ventricular function and hypertrophy parameters, supporting the hypothesis of a myocardial and systemic inflammatory reaction going in hand with mild cardiac hypertrophy and preserved heart function at this stage of AS. In sham animals, a higher number of negative r-values was observed compared with WI.

**Figure A6:**
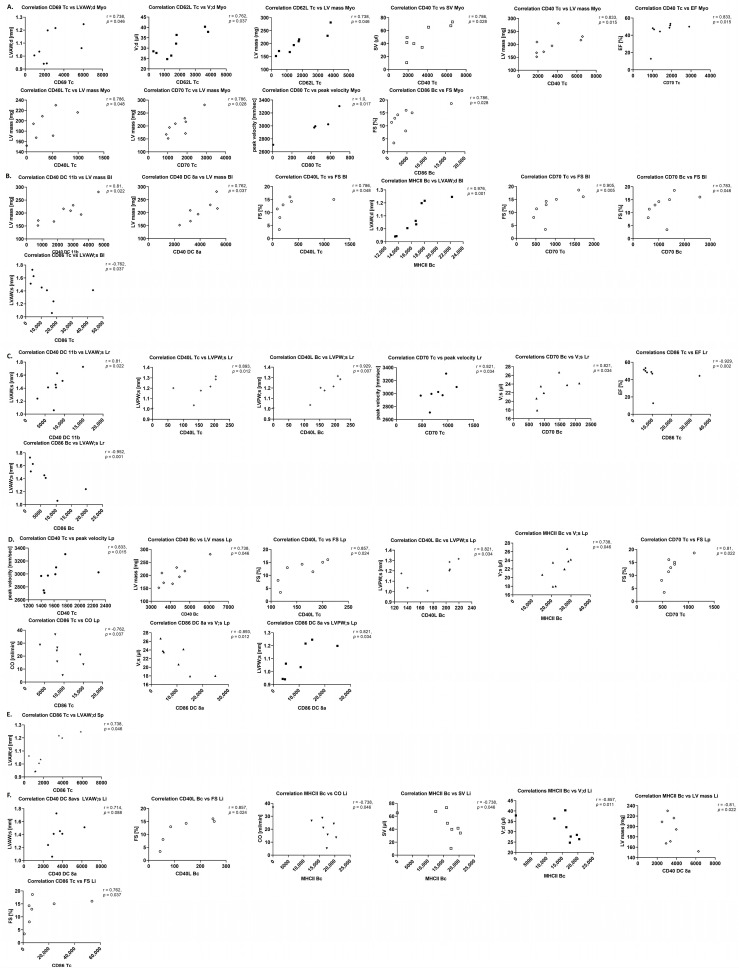
Costimulatory marker expression on T cells, B cells and DCs in heart, blood immune cells and peripheral immune organs upon AS assessed with flow cytometry. On T cells, B cells and DCs isolated from (**A**) heart, (**B**) blood, or peripheral immune organs ((**C**) regional lymph nodes, (**D**) peripheral lymph nodes, (**E**) spleen, (**F**) liver), expression of costimulatory markers was measured with flow cytometry. The Spearman correlation analysis between costimulatory molecules on the respective immune cell type, and echocardiographic parameters was performed for WI animals. Displayed are graphs of significantly correlated parameters. Data are values of *n* = 8 WI animals; *p* < 0.05 was considered statistically significant.

Also, for correlations of immune organs with myocardium, most r-values were negative (mainly trending towards up to significant correlation) for CD80 (except of liver) and CD86 (except of blood), a phenomenon that was even stronger in sham, where it was found in all organs for CD80 and in spleen and liver for CD86 (Figure 6B and Figure A6).

In ^1^H/^19^F MRI, which is used for non-invasive inflammation imaging, intravenously injected perfluorocarbon nanoemulsions (PFCs) serve as nanotracers, which accumulate in inflammatory foci after being taken up by phagocytic immune cells in the peripheral blood. This study, analyzing in vitro phagocytosis of PFCs by immune cells as a readout of cell activation state, reveals a significantly reduced PFC uptake by both T and B cells upon AS (Figure 6C), supporting the hypothesis of a chronic systemic immune reaction during this disease.

## 4. Discussion

In the presented study, the systemic inflammatory reaction upon murine WI and the link between local processes and the systemic inflammatory reaction were investigated. The results suggest a local aortic and myocardial, and a systemic inflammatory reaction, which besides of a chronic also have an acute component, and supposedly are mechanistically linked with AS severity, preserved heart function, and mildly altered myocardial structure four weeks after WI surgery. Remarkable were hints at a T cell recruitment from peripheral immune organs and liver into the aortic valve. However, also in sham, an inflammatory reaction was found which was associated with blood flow velocity over the aortic valve and with lower heart function.

### 4.1. Stenotic Aortic Valves Present with an Inflammatory Reaction Closely Associated with AS Severity and Left Ventricular Function and Structure

Amongst the immune cells which transmigrate into the aortic valve parenchyma upon AS are T cells as main immune cell type in the final stage of AS and B cells, which are not present in the normal cardiac valve, but upon AS are associated with valve calcification and the maximum transvalvular gradient [34]. This is in line with increased numbers of T cells in the aortic valve upon WI, while B cell numbers were unchanged but closely correlated with left ventricular mass in this study.

In the areas of inflammatory cell infiltrates within the fibrocalcific valves, cytokines are produced by T cells and macrophages [5]. This study shows an increase in pro-inflammatory *IL-6*, which is known to be responsible for T and B cell activation, T cell differentiation and lymphocyte tissue infiltration [35,36]. In addition, a trend towards increase in anti-inflammatory *IL-10* was observed, which suppresses T cell activation [37] but induces immunoglobulin and cytokine production by B cells [38], and migration of CD8^+^ T cells [39], while inhibiting migration of B cells [38]. *TNF-α* levels, which can act both pro- and anti-inflammatory and play an important role in the regulation of the immune reaction, but also tissue degradation and regeneration [40] trended to be reduced in this model, which might hint at a dysregulation of the immune system within the aortic valve upon AS at this time point. Most importantly, cytokines in the aortic valve showed several positive r-values for correlation with echocardiographic parameters, in contrast to more negative r-values in sham animals, underlining the influence of the local inflammatory reaction in the aortic valve, resulting, e.g., in remodelling of the valve tissue, on AS severity and left ventricular function and structure.

### 4.2. A Local Inflammatory Reaction Correlating with AS Severity and Left Ventricular Function and Structure Is Found in the Myocardial Tissue upon AS

The mild myocardial hypertrophy observed in this model of moderate AS [20] has been associated with myocardial inflammation also in other experimental studies, as well as in patients with AS. In patient tissue, cell densities of macrophages and newly recruited leukocytes were higher in the AS group, which together with increased expression of chemokines and suppression of anti-inflammatory *IL-10* indicated a persistent and predominantly chemokine-driven inflammatory reaction, while mRNA levels of *TNF-α* or *IL-1β* were unchanged [8].

This is in line with the observation of trends towards correlation for immune cell numbers in the myocardium with echocardiographic parameters in this study, while in contrast to literature, immune cell numbers were unchanged, and trends towards an increase in cytokine levels investigated with RT-qPCR (*IL-6*, *TNF-α*, *IL-10*) were observed.

The observation of higher levels of most costimulatory molecules on lymphoid cells and DCs, although mostly not trending towards significance, but in some cases significantly correlated or trending towards correlation with echocardiographic parameters, as well suggests stress-induced activation of immune cells due to cardiac pressure overload, as described in the literature [9].

### 4.3. Local and Systemic Levels of Immune Cells Are Altered and Related to Left Ventricular Function and Structure and AS Severity

Local inflammatory processes can induce systemic inflammatory reactions as migration of immune cell populations in the form of inflammation-mediated emptying of leukocyte reservoirs to serve the increased need in the inflammatory focus [27], or cytokine-mediated leukocyte retention within organs [26]. This study showed trends towards reduced numbers of T cells in spleens, livers, and regional and peripheral lymph nodes upon AS. Changes in immune cell numbers in the different organs showed correlations up to significance with left ventricular echocardiographic parameters upon AS—remarkable were the positive correlation of B cell numbers in the aortic valve with echocardiographic parameters, and positive and negative r-values for the correlation with AS severity and left ventricular function or hypertrophy parameters, respectively, for regional lymph nodes. The pattern of correlations was more heterogeneous in sham animals.

These observations are in line with a model of cardiac pressure overload, in which immune cell recruitment from the periphery into the myocardium was shown to play an important role in the local inflammatory reaction in the heart. Here, inhibition of PI3K-γ activity in bone marrow cells reduced leukocyte infiltration into the myocardium upon pressure overload, resulting in reduced cardiac inflammation, remodelling, and preservation of cardiac function [7].

### 4.4. A Systemic Chronic Inflammatory Reaction Associated with AS Severity and Left Ventricular Function and Structure Is Present upon AS

For a more detailed analysis of the link between the systemic inflammatory reaction and the inflammatory processes in the heart upon AS, this study took a closer look at cytokine and costimulatory molecule expression in myocardium, blood and peripheral organs, and CD4/CD8 T cell ratio in blood and myocardium, and uptake of nanoemulsions by peripheral blood immune cells.

A differential up- and down-regulation of costimulatory molecules and cytokines at mRNA level, displaying multiple trends towards and correlations with AS severity and left ventricular echocardiographic parameters was observed, with a more homogeneous correlation pattern upon AS compared with sham animals. Cytokines at protein level, in contrast, were unaltered between sham and WI animals and showed only a small number of trends towards correlation; however, r-values were mostly positive for both myocardium and blood, which was different from sham animals.

Two mechanisms might cause the systemic inflammatory reaction upon AS: circulating immune cells may be activated by shear forces while passing the stenotic aortic valve [41] or by factors within the myocardium stressed by, e.g., pressure overload upon AS [42].

In an in vitro microfluidics system, in accordance with the first hypothesis, Baratchi et al. showed increased mRNA levels of *IL-6* and *IL-1β* in monocytes subjected to shear stress, while *TNF-α* and *IL-10* levels remained unchanged [41]. Stressed cells besides showed other signs of activation, as increased adhesion to stimulated endothelial cells and increased phagocytosis [41].

However, also in the presence of a healthy aortic valve, a systemic inflammatory reaction is found upon cardiac hypertrophy, as observed in resistance hypertension. The systemic inflammatory reaction in this disease presents with increased systemic cytokine and acute phase protein levels. A difference between this heart disease and AS is the accompanying inflammation also in the atherosclerotic endothelium [10,43,44]. But also upon hypertrophic cardiomyopathy (HCM) of other causes, systemic cytokines and acute phase proteins are increased, while levels of circulating inflammation markers were directly associated with cardiac remodelling [11].

As co-stimulation occurs together with antigen presentation prior to clonal expansion and subsequent homing of T cells to inflammatory foci [15], the observations in the present study support the notion of a recruitment of activated peripheral T cells to the heart upon AS.

Leukocyte numbers in the blood, e.g., numbers of myeloid cells, were found to be increased in HCM patients and to be correlated with cardiac remodelling and diastolic dysfunction [11]. In addition, in a recent study by Hoffmann et al., increased baseline acute phase protein and IL-6 levels and numbers of Th17 cells, but reduced numbers of Th2 cells, were observed as markers of reduced 12-month survival after transcatheter aortic valve replacement (TAVR) [6]. This suggests the systemic inflammatory reaction as an important prognostic marker, underlining the clinical relevance of the present study.

The reduced uptake of PFCs by blood-derived lymphoid cells isolated from WI animals compared with sham may be used as a prognostic readout both in vitro, as well as, upon transfer of this imaging technique into the clinic, in vivo [6,45].

### 4.5. Novelty and Significance

Assessing the local processes in the heart, this study contributes to the understanding of the inflammatory reaction within the aortic valve upon AS and its contribution to AS severity and influence on left ventricular function and structure. The study adds information to the knowledge about stress-induced activation of myocardial immune cells upon cardiac pressure overload, which induces the secretion of inflammation mediators. What is besides new, is the correlation of peripheral cytokine levels and expression levels of costimulatory molecules on cells of the adaptive immune system in the peripheral immune organs and the blood with disease severity and left ventricular function and hypertrophy also in this murine model of AS.

These observations support the hypothesis of an activation of a systemic immune reaction and its close mechanistic link, e.g., by recruitment of peripheral immune cells after co-stimulation, to processes in the heart.

The knowledge gained in the present study is the basis of understanding the complex systemic interplay of inflammatory processes with heart function and hypertrophy in the presence of AS, which if it is better studied may offer checkpoints at which a therapy can be applied to improve patient management.

### 4.6. Limitations

This study investigates an early state of AS induced by WI without any cardiovascular risk factors such as hypertension, diabetes or dyslipidemia [46]. However, AS patients regularly suffer from such diseases, which impact on processes in the aortic valve, on heart function and on inflammatory reactions and may as well impact on their interplay. Therefore, in future studies on this mouse model of AS, comorbidities should be addressed—which in this model is relatively easy as it allows to use different strains or ages, and to combine the surgery with dietary or medical interventions. This will serve to find out how treatment of the comorbidities impacts on the complex systemic effects of AS, including the systemic immune reaction, cardiac hypertrophy, and the worsening of heart function [47,48].

A limitation of this study is that experiments were only conducted in male animals. This was meant to initially reduce animal numbers in accordance with 3R (replace, reduce, refine), as an influence of female hormones on inflammation is known, which may increase the inter-individual variation, that is, the standard deviation amongst female mice [49]. Therefore, this study is not representative of both sexes and now that a systemic inflammatory reaction upon AS has been found, subsequent studies will have to be performed in mice of both sexes.

As another limitation, although the murine AS model very well reproduces the human disease, transfer of data from mice to men is limited, wherefore results cannot be generalized and must be reproduced in human-derived material.

Echocardiography in this study was performed from two weeks after WI on, to assess successful induction of the AS, as already one week after surgery, an increase in peak blood flow velocity over the aortic valve can be detected as sign of a successful induction of the pathophysiology [19]. However, an early AS with the characteristic signs in the aortic valve and reduced heart function is only found after four weeks, the time point at which the immune cell and molecular assessments were performed. This justifies that experiments on a cellular and molecular level were only conducted cross-sectionally at one time point four weeks after WI. To assess the processes during the progression of the disease, a longitudinal study will have to be conducted, comprising also later time points.

Additionally, due to small tissue volumes especially of the aortic valve, material from single animals could only be used for one assay, preventing the analysis of correlation of data between different modalities within one animal. However, as echocardiography was performed in vivo in all animals prior to sacrifice and does not require to isolate tissue samples, it was possible to correlate the results of ex vivo assays with echocardiographic parameters of heart function and hypertrophy. The problem of small samples in the future can only be circumvented by using either larger animal species as rabbits and pigs [50], or by using patient samples.

Furthermore, signs of a systemic inflammatory reaction in sham animals, mirrored by differences in cytokine levels between sham and untreated control animals, suggest an effect of the mere surgical procedure (skin incision, incision of one carotid artery, endothelial damage due to advancing the wire, carotid artery ligation), independent of the development of AS, on the inflammatory reaction. To eliminate this confounding factor, in all experiments, sham groups were used.

## 5. Conclusions

As a novel finding, this study detected a local myocardial and a systemic inflammatory reaction in the WI mouse model of AS four weeks after surgery, comprising changes in immune cell numbers and ratios and cytokine and costimulatory molecule expression on adaptive immune cells. The results newly suggest that the local cardiac inflammatory reaction triggers that in the periphery, herewith recruiting immune cells to the inflamed heart. As immune cells in the myocardium induce cardiac remodelling, the systemic inflammatory reaction therefore is supposed to have a direct impact on heart function upon AS, in this study underlined by the correlation of local and peripheral inflammatory processes with AS severity and left ventricular function and hypertrophy.

With myocardial remodelling as a major determinant of malignant arrhythmias and end-stage systolic heart failure in HCM [11], the aim of future studies should be to find out if therapeutic modification of the inflammatory reaction, potentially by treating comorbidities, improves heart function, and herewith reduces heart failure rate in AS patients.

## Figures and Tables

**Table 1:** Power calculation.

Method	Mean CTRL	Mean EXP	SD CTRL	SD EXP	POWER	*n*/Group
Echocardiography	63.6	90.3	10.1	20.8	0.8	6
Biochemical analysis (Inflammation)	550.6	339	182.3	76	0.8	7
Cytokines blood	9.3	23.7	7.2	10.9	0.8	8
Flow cytometry	29	7	42	8	0.8	7
Molecular biology	0.4	0.55	0.1	0.1	0.8	9
^19^F-MRT	191	231	39.6	59.2	0.8	10

CTRL: Control group; EXP: experimental group; SD: standard deviation.

**Figure A1:**
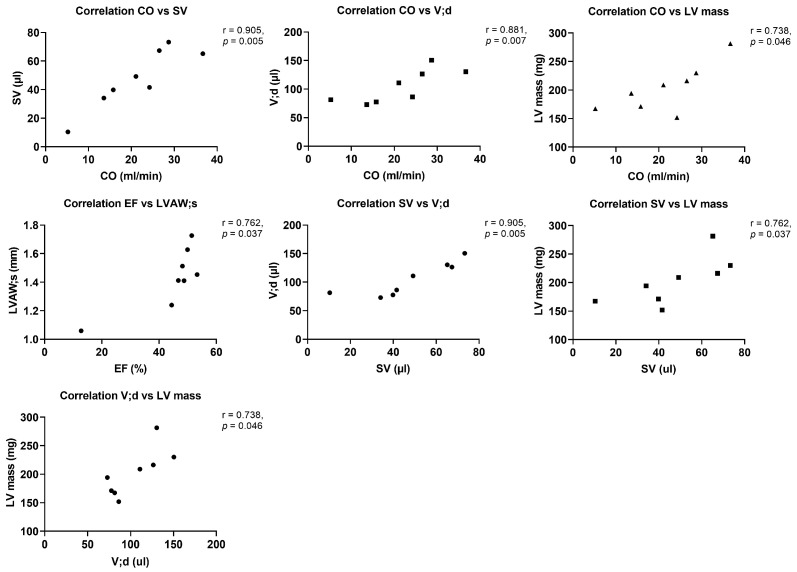
Assessment of AS development and heart function with echocardiography. Echocardiography at baseline, one and four weeks after WI was used to assess severity of AS and left ventricular function and structure. The Spearman correlation analysis between echocardiographic parameters was performed for WI animals four weeks after surgery. Displayed are graphs of significantly correlated parameters. Data are values of *n* = 8 WI animals; *p* < 0.05 was considered statistically significant.

**Figure A3:**
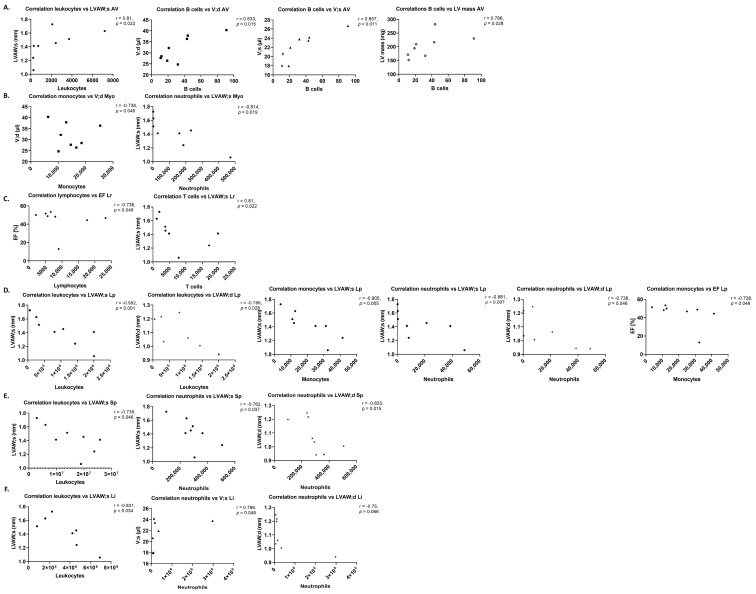
Immune cell numbers in the heart and peripheral immune organs upon AS. Four weeks after surgery, total number of immune cells per explanted (**A**) aortic valve, (**B**) myocardium, (**C**) regional lymph node, (**D**) peripheral lymph node, (**E**) spleen or (**F**) liver was measured with flow cytometry. The Spearman correlation analysis between total immune cell numbers of organs and echocardiographic parameters was performed for WI animals. Displayed are graphs of significantly correlated parameters. Data are values of *n* = 8 WI animals; *p* < 0.05 was considered statistically significant.

## Data Availability

The raw data supporting the conclusions of this article will be made available by the author on request.

## Abbreviations

The following abbreviations are used in this manuscript:

AI	Aortic valve insufficiency
APC	Antigen presenting cell
AS	Aortic valve stenosis
AV	Aortic valve
BSA	Bovine serum albumin
BW	Body weight
cDNA	Complementary deoxyribonucleic acid
CO	Cardiac output
CTRL	Control group
DAPI	4′, 6-Diamidino-2-phenylindole
DC	Dendritic cell
DMEM	Dulbecco’s modified eagle’s medium
ECG	Electrocardiogram
ECM	Extracellular matrix
EDTA	Ethylenediaminetetraacetic acid
EF	Ejection fraction
EXP	Experimental group
FACS	Fluorescence associated cell sorting
FcR	Fragment crystallizable receptor
FS	Fractional shortening
HCM	Hypertrophic cardiomyopathy
Lnn per	Peripheral lymph nodes
Lnn reg	Regional lymph nodes
LV	Left ventricle
LVAW;d	end-diastolic left ventricular anterior wall
LVAW;s	end-systolic left ventricular anterior wall
LVPW;d	end-diastolic left ventricular posterior wall
LVPW;s	end-systolic left-anterior posterior wall
MACS	Magnetic assisted cell sorting
MFI	Mean fluorescence intensity
MI	Myocardial infarction
Myo	Myocardium
mRNA	Messenger ribonucleic acid
PBS	Phosphate-buffered saline
PFC	Perfluorocarbone nanoemulsion
RT-qPCR	Quantitative real-time polymerase chain reaction
SD	Standard deviation
SV	Stroke volume
TAVI	Transcatheter aortic valve intervention
TAVR	Transcatheter aortic valve replacement
Tc	T cells
VEC	Valvular endothelial cells
VIC	Valvular interstitial cells
WI	Wire injury surgery
wks	weeks
ZETT	Zentrale Einrichtung für Tierforschung und wissenschaftliche Tierschutzaufgaben
